# Tracking Algorithm of Multiple Pedestrians Based on Particle Filters in Video Sequences

**DOI:** 10.1155/2016/8163878

**Published:** 2016-10-25

**Authors:** Hui Li, Yun Liu, Chuanxu Wang, Shujun Zhang, Xuehong Cui

**Affiliations:** School of Information Science and Technology, Qingdao University of Science and Technology, Qingdao 266000, China

## Abstract

Pedestrian tracking is a critical problem in the field of computer vision. Particle filters have been proven to be very useful in pedestrian tracking for nonlinear and non-Gaussian estimation problems. However, pedestrian tracking in complex environment is still facing many problems due to changes of pedestrian postures and scale, moving background, mutual occlusion, and presence of pedestrian. To surmount these difficulties, this paper presents tracking algorithm of multiple pedestrians based on particle filters in video sequences. The algorithm acquires confidence value of the object and the background through extracting a priori knowledge thus to achieve multipedestrian detection; it adopts color and texture features into particle filter to get better observation results and then automatically adjusts weight value of each feature according to current tracking environment. During the process of tracking, the algorithm processes severe occlusion condition to prevent drift and loss phenomena caused by object occlusion and associates detection results with particle state to propose discriminated method for object disappearance and emergence thus to achieve robust tracking of multiple pedestrians. Experimental verification and analysis in video sequences demonstrate that proposed algorithm improves the tracking performance and has better tracking results.

## 1. Introduction

Object tracking [1, 2] is an important research field in computer vision for its wide range of application demands and prospects in industries, such as intelligent human-computer interaction, video monitoring, and intelligent transportation. Pedestrian is the main goal of tracking in most scenes of object tracking. Pedestrian tracking [3] has important research significance and application value in object tracking. However, multiple pedestrians tracking in complex environment is still facing many problems due to randomness in human motion, pedestrian scale changes and posture changes, mutual occlusion, complex backgrounds, and so forth.

For video object tracking study, there are mainly three methods: (1) method based on pattern matching, (2) method based on classification, and (3) method based on object state estimation. Method based on pattern matching is the method which transforms visual tracking into object matching of successive video frames [4]. Mean Shift [5, 6] is the most typical object pattern matching algorithm. This method has relatively small calculating amount and can achieve fast pedestrian detection and tracking in static background. However, it is difficult for pedestrian detection and tracking in moving background, which limits the application range of this method; method based on classification [7–9] transforms object tracking into foreground and background classification and usually adopts machine learning method for processing. But there are three problems of this method: first, construction of classifier needs a large amount of positive and negative samples to learn and how to choose samples is a key issue; second, the calculation has high complexity and large calculation amount; thus it is hard to satisfy real-time needs; third, it needs to do object search within the scope of object region. It still needs to study how to optimize the scope to the size which is neither too small to affect object tracking precision nor too large to reduce searching efficiency. Method based on object state estimation [10] is based on Bayesian theory. The method achieves object tracking by iteratively solving the maximum posterior probability of object state under new observation value condition on the basis of acquiring prior probability of object state. For its stable tracking performance and the continuous improvement of computer processing ability in recent years, it has been widely used in video tracking. Due to particle filters' non-Gaussian, nonlinear assumption and multiple hypothesis property, it has been successfully applied to video object tracking [11]. Therefore, it becomes the mainstream research method [12].

## 2. Previous Work

Since people differ from other objects, they belong to nonrigid objects, and they have various shapes and deformations; it is difficult to describe their features. Compared with single pedestrian tracking, multiple pedestrians tracking is more complex. It needs to estimate the state of multiple pedestrians and is difficult to deal with similar appearance, mutual block, disappearance, and appearance among pedestrians. Isard et al. [13] proposed Bayesian Multi-Blob tracker on the basis of a combination of blob algorithm and particle filter algorithm. The tracker can handle number changes of objects during the process of tracking but only for a static background. It is difficult to construct the observation model when the background is moving. Okuma et al. [14] proposed an enhanced particle filter which can achieve tracking of ice hockey players. The method combines the two successful algorithms, hybrid particle filter and AdaBoost, to enable automatic multiobject tracking system. However, it is prone to tracking failure when the background is comparatively more complicated; Henriques et al. [15] proposed a new theoretical framework, CSK, for analyzing sample in detection and tracking. The method can quickly and accurately train trackers which can be used to track pedestrians. Danelljan et al. [16] improved CSK method and adopted self-adaptive dimensionality reduction method to extract color feature which improves CSK's real-time effect in tracking pedestrian, but CSK is based on classification, and, with object increasing, the efficiency and the real-time effect will also be reduced; Lu et al. [17] combined random airport tracking with DPM pedestrian detection and corrected detection result by mutual assistance between them and calibrated NBA basketball players through the feature extraction and recognition. Chen et al. [18] proposed constrained sequential labeling (CSL), which can be used for volleyball and basketball player tracking. However, both of the above two methods can only track indoor scenes, which are rarely affected by light, weather, and other complex factors. Therefore, they are not suitable for tracking pedestrians in complex outdoor scenes; Yang et al. [19] proposed a robust superpixel tracking method. The method fully takes advantage of intermediate visual features and constructs object tracker based on superpixel to accommodate the nonrigid deformation and out-of-plane rotation of the object. Thus, it has better robustness for nonrigid deformations, illumination changes, big posture changes, and out-of-plane rotations. The method can be extended to multiple pedestrians tracking; however, it only considers the superpixel color information; thus it is prone to cause tracking errors when being applied to multiple pedestrians tracking. Xue et al. [20] consider the problem of human tracking in RGBD videos filmed by sensors such as MS Kinect and Primesense. This method can achieve indoor multipedestrian tracking quickly and accurately. Since the distance that Kinect requires to collect object should be controlled between 1.6 m and 3 m, the pedestrian beyond 3 m cannot be tracked. Furthermore, this method is susceptible to occlusion. Wu et al. [21] proposed a regional deep learning tracker that observes the object by multiple subregions and each region is observed by a deep learning model. However, with the increase of tracked objects, it can cause a huge deep network; thus it is difficult to achieve efficient tracking.

In summary, the major issues to be addressed for the current multipedestrian tracking based on particle filter are as follows: (1) tracking under complex situations such as pedestrian posture, dimension, and other changes and the temporary disappearance of object caused by severe or completely occlusion; (2) multiple pedestrians tracking under a moving background; (3) pedestrian disappearance and appearance in multiple pedestrians tracking caused by the limited range of camera shooting; (4) the fact that, for multiple pedestrians tracking, mutual occlusion and interference among pedestrians often occur. Therefore, effective tracking algorithm is needed to reduce errors of object tracking results and track the real state and trajectory of the object.

**Figure 1:**
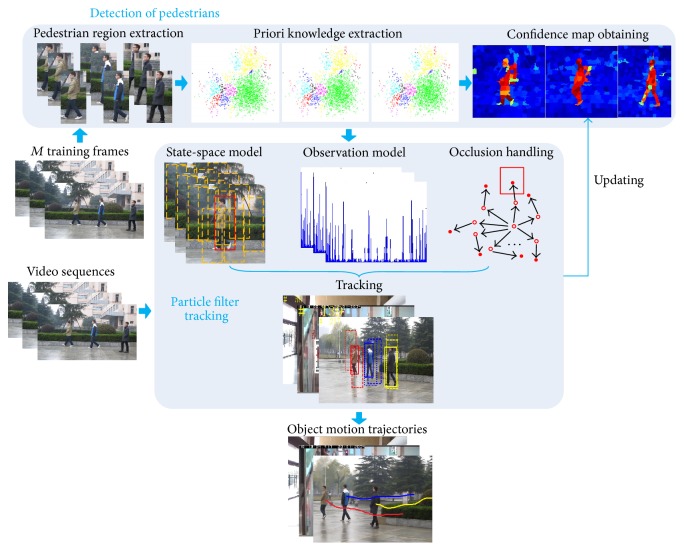
Diagram of algorithmic process.

In order to solve the above problems and achieve automated and robust tracking of pedestrians in complex scenarios, we present tracking algorithm of multiple pedestrians based on particle filters in video sequences.  Figure 1 shows diagram of algorithmic process.

## 3. Detection of Pedestrians

### 3.1. Object Region Extraction

**Figure 2:**
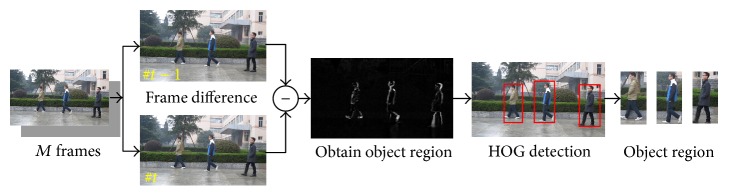
Flow diagram of object regions extraction of *M* frames.

Before tracking, we need to detect object regions in the first *M* frames. If the value of *M* is too small, it will affect the accuracy of a priori knowledge; otherwise it will affect the efficiency of tracking algorithm. Therefore, after weighing between efficiency and accuracy, we set the number of *M* frames as 5 through experimental testing. In order to get object region, there are two main processes: firstly, adopt frame difference method to obtain the object region; then obtain detection results by using HOG detector.  Figure 2 shows the flow diagram of object regions extraction of the first *M* frames. This detection method not only reduces HOG detection region but also improves efficiency and accuracy of the detection.

### 3.2. A Priori Knowledge Extraction

Enlarge object region detected by image of *M* frames and do superpixel segmentation in rectangular region which is larger than the object region thus to get a priori knowledge which reflects the object and background. Description of prior knowledge acquisition process is as follows:

(1) Define two rectangular regions *R*_*t*_^1^, *R*_*t*_^2^ for *t* frames, *R*_*t*_^1^ represents detected object region of the last frame, and *R*_*t*_^2^ represents rectangular region whose side lengths are, respectively, 1.5 times the maximum length and width of object region, that is, a rectangular region which is slightly larger than object region *R*_*t*_^1^. *R*_*t*_^2^ − *R*_*t*_^1^ represents the middle region of two rectangles, that is, the nonoverlapping part of two regions.
(2) Do superpixel segmentation for *R*_*t*_^2^ region; if superpixel(*i*, *j*) is superpixel at position (*i*, *j*), then *f*_*t*_^sp^ represents the spth feature vector of superpixel. Feature vector here is HSV color histogram.
(3) Employ Mean Shift clustering for superpixel feature vector *F* = {*f*_*t*_^sp^∣*t* ≥ 1; sp = 1,2,…, sp_num} and get cl_num different clusters.
(4) In feature space, each cluster contains the following information: cluster center *C* = {*C*_cl_}_cl=1_^cl_num^, cluster radius radius(*i*), and cluster member {*f*_*t*_^sp^∣*t* ≥ 1; *f*_*t*_^sp^ ∈ cluster(*i*)}. Thus, each cluster corresponds to a certain region in image frame. Compute two areas for the cluster: area^ob^(*i*) and area^bg^(*i*). area^ob^(*i*) represents the area occupied by cluster *i* in object region *R*_*t*_^1^; area^bg^(*i*) represents the area occupied by cluster *i* in object region *R*_*t*_^2^ − *R*_*t*_^1^. Thus, the larger area^ob^(*i*) − area^bg^(*i*) value is, the more superpixel members of cluster *i* appear in the object region; and the smaller area^ob^(*i*) − area^bg^(*i*) value is, the more superpixel members of cluster *i* appear in the background region. According to this condition, we use the following method to calculate confidence value for each cluster in region [−1,1]:
  (1) $$\begin{array}{c} C_{i}^{F}=\frac{area^{ob}\left(i\right)-area^{bg}\left(i\right)}{area^{ob}\left(i\right)+area^{bg}\left(i\right)},\;i=1,2,\ldots ,{cl}_{num}. \end{array}$$

**Figure 3:**
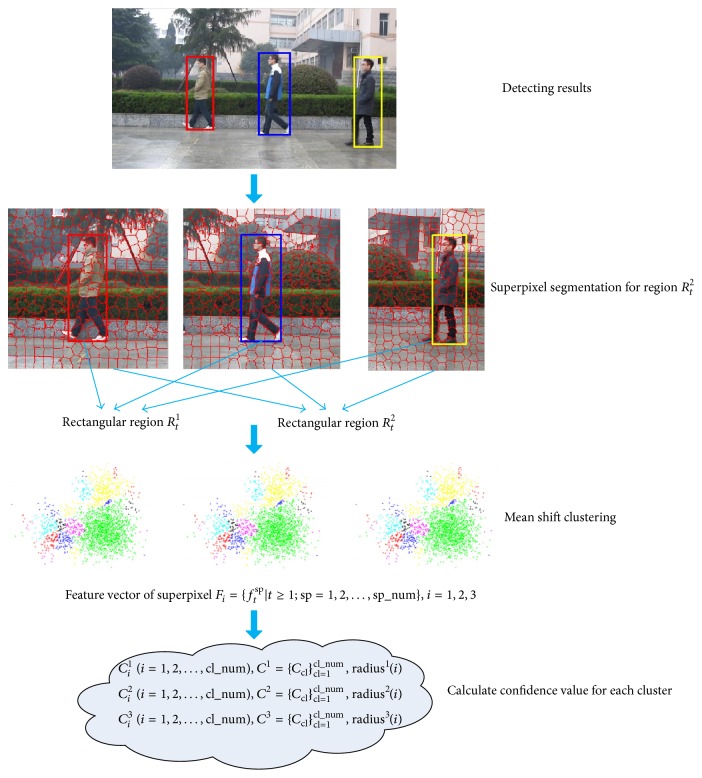
A priori knowledge acquisition process.

Establish object and background prior knowledge based on the object region obtained from the first *M* frames. The process of acquiring a priori knowledge is shown in Figure 3.

### 3.3. Confidence Map Obtaining

When a new frame, that is, *M* + 1 frame, arrives, do superpixels segmentation in the region which is 1.5 times object region based on the location of the last frame and acquire *N*_*t*_ superpixels and then calculate the object/background confidence value for each superpixel according to the following formula:

(2) Wsp,i=e−λdfisp−fci/rci,sp=1,2,…,Nt;  i=1,2,…,clnum,Csps=Wsp,i·CiF,sp=1,2,…,Nt,

where *W*(sp, *i*) is weighting term, which measures the distance between the spth superpixel in the *t*th frame and cluster center *f*_*c*_(*i*) which it belongs to. The closer the distance is, the closer confidence value of the superpixel is to the object confidence value of the cluster, and vice versa. Parameter *r*_*c*_(*i*) represents radius of cluster *i*, and *λ*_*d*_ is a normalization term, used to adjust distinction degree of superpixel weight in clustering. The choice of this value was made in light of existing research literature and the fact that the optimal value for the threshold should lie near the bend of the ROC curve. This was consistent across a number of trials. Thus, by referring to previous studies, we set lambda *d* as 2. According to the above method, object/background confidence value for each superpixel *C*_sp_^*s*^ in each new frame can be calculated by using cluster confidence value *C*_*i*_^*F*^ and the distance weight item *W*(sp, *i*).

**Figure 4:**
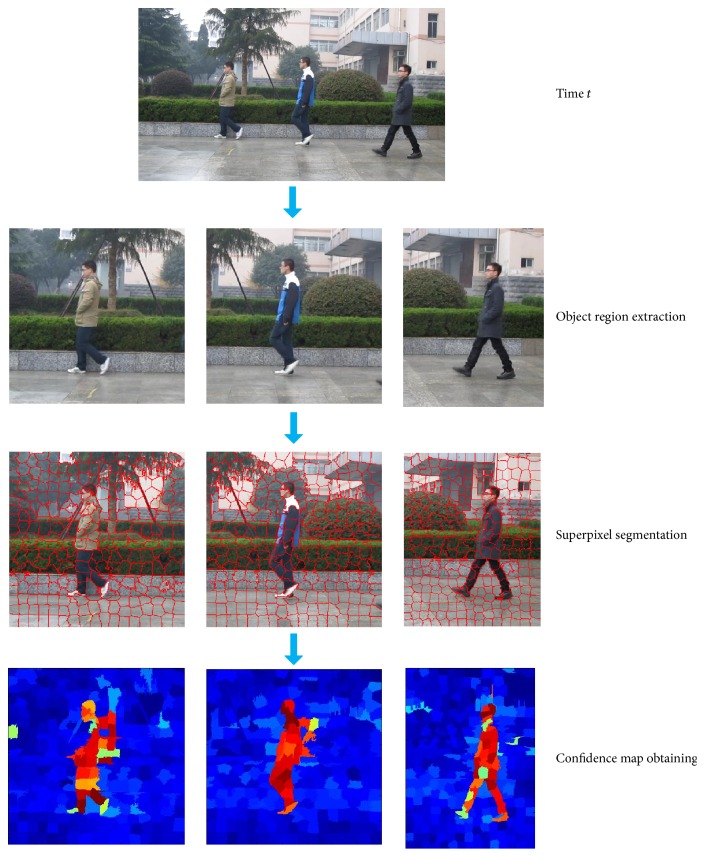
The *t*th frame confidence map acquisition process.

Finally, assign each pixel of superpixels in the frame of the superpixel confidence value *C*_sp_^*s*^, and assign pixel point in region outside superpixel segmentation confidence −1. The confidence values of all pixels in the current frame are obtained, and thus confidence map of the current frame is obtained. The entire process is shown in Figure 4, where, in the confidence map, deeper blue represents the greater possibility of superpixel to be belonging to the background, and deeper red represents the greater possibility of the superpixel to be belonging to the object.

**Figure 5:**
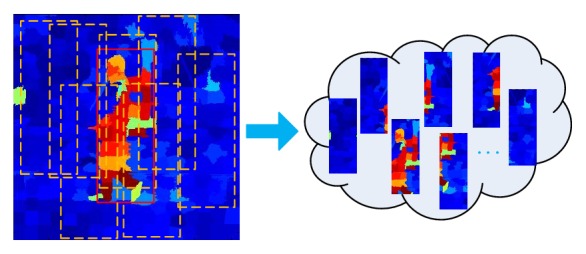
Random sampling of object confidence map.

After acquiring object confidence map of the current frame, randomly sample *N* image blocks in rectangular region *R*_*t*_^2^. The size of the block image is the same as the object rectangular region *R*_*t*_^1^ of the previous frame, as shown in Figure 5. Then calculate confidence values of pixel points in the locations of each sampling image block center. The image block which has the greatest confidence value is detection result.

## 4. Particle Filter Tracking

Nonlinear, non-Gaussian distribution system can more accurately describe pedestrian tracking in actual complex scenes, and particle filter algorithm can handle any nonlinear, non-Gaussian distribution systems. For the above reason, we choose particle filter framework to solve pedestrian tracking in complex scenes.

### 4.1. State-Space Model

Provided *X*_*t*_ = [*x*_1,*t*_, *x*_2,*t*_,…, *x*_*F*,*t*_], *F*_*t*_ is the state number in *X*_*t*_, *F*_*t*−1_ is the state number in *X*_*t*−1_, *x*_*j*,*t*_ is the object state of the *j*th object at time *t*, object state is *x*_*j*,*t*_ = [*x*_*j*_, *y*_*j*_, *θ*_*j*_, *s*_*j*_] during the test process of object tracking, state of the object is indicated as *x*_*j*,*t*_ = [*x*_*j*_, *y*_*j*_, *w*_*j*_, *h*_*j*_], *x*_*j*_ and *y*_*j*_ are, respectively, the coordinates of rectangle center at direction *x* and direction *y* in the image, and *θ*_*j*_ and *s*_*j*_ are, respectively, width and height of the rectangle. Since each object motion is an independent process, joint product of a single object model can be used to track multiple objects:

(3) $$\begin{array}{c} p\left(\;X_{t}\;|\;X_{t-1}\;\right)=\overset{F}{\underset{i=1}{\prod }}p\left(\;x_{i,t}\;|\;x_{i,t-1}\;\right). \end{array}$$

In order to obtain the state transition density function of the *j*th object at time *t*, we use stochastic disturbance model to describe state transition of the *j*th object from time *t* − 1 to time *t*, which is shown as the following formula:

(4) $$\begin{array}{c} p\left(\;x_{j,t}\;|\;x_{j,t-1}\;\right)=N\left(\;x_{j,t};x_{j,t-1},\Sigma \;\right), \end{array}$$

where *N*(*x*_*j*,*t*_; *x*_*j*,*t*−1_, Σ) indicates normal density function, the covariance of which is diagonal matrix Σ, and the elements on the diagonal correspond to variances of four parameters in state *x*_*j*,*t*_, namely, *σ*_*x*_^2^, *σ*_*y*_^2^, *σ*_*θ*_^2^, *σ*_*s*_^2^, respectively, representing variance of object center coordinates, width, and height of the rectangle.

**Figure 6:**
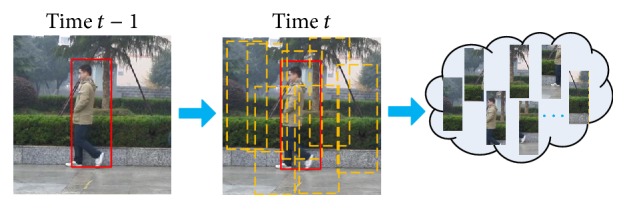
Sampling of candidate regions.

According to the object position of the last time, acquire particles of each object by state-space model sampling; each particle represents a candidate region.  Figure 6 shows the sampling of candidate regions. Then, calculate observation value of each candidate region at the position pixel by observation model.

### 4.2. Observation Model

Do feature extraction of acquired *F* object detection results at the current time to obtain observation value of the current object *Z*_*t*_ = {*z*_1,*t*_, *z*_2,*t*_,…, *z*_*F*,*t*_}; observation value of *P* predicted particles is *y*_*t*_ = {*y*_*t*_^1^, *y*_*t*_^2^,…, *y*_*t*_^*P*^}_1_^*N*^. In observation model, observation value of particles and that of object need to be correlated to select the best particle, and candidate region represented by the particle can be seen as detection result.

#### 4.2.1. Feature Extraction

**Figure 7:**
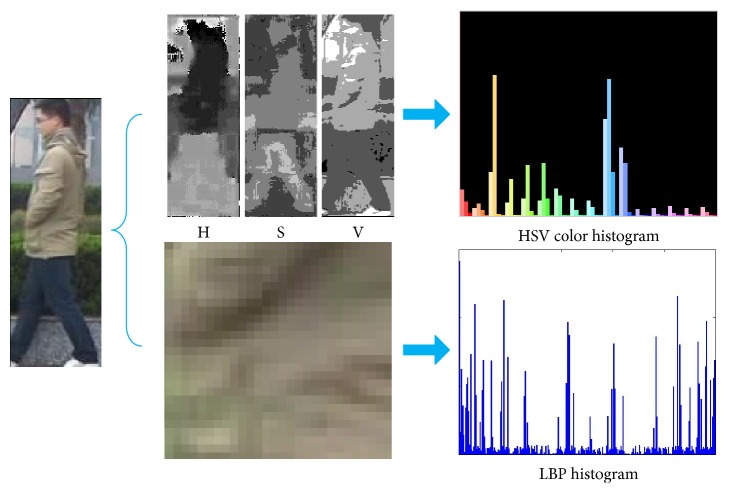
Diagram of feature extraction.

In order to obtain the observed values of object and particles, respectively, feature extraction needs to be done for detected regions and candidate regions. Tracking method based on a single feature can get better tracking results in some special scenarios or situations with little changes in the object environment, and so forth. However, using this method always leads to loss of tracking under more complex environment, background, or the influence of noise, likeness interference, and other factors. In such cases, using multifeature combination is able to get a better applicability. Therefore, this paper adopts two features which are complementary to each other, HSV color histogram and LBP histogram, as shown in Figure 7.

#### 4.2.2. Association of Particles and Observed Values

After the state transition of particles based on the equation of state, particle state at the new time *t* can be acquired. Set the object state number at *t* time as *N*_*t*_; *N*_*t*_ object state particle sets can be expressed as

(5) $$\begin{array}{c} S_{t}=\left[\begin{array}{cccc} x_{1,t}^{1} & x_{2,t}^{1} & \cdots & x_{N_{t},t}^{1}\\ x_{1,t}^{2} & x_{2,t}^{2} & \cdots & x_{N_{t},t}^{2}\\ \vdots & \vdots & \cdots & \vdots \\ x_{1,t}^{P} & x_{2,t}^{P} & \cdots & x_{N_{t},t}^{P} \end{array}\right]. \end{array}$$

Prediction observation value of *P* particles corresponding to its object state is *y*_*t*_ = {*y*_*t*_^1^, *y*_*t*_^2^,…, *y*_*t*_^*P*^}_1_^*N*^; the current object observation value is *Z*_*t*_ = {*z*_1,*t*_, *z*_2,*t*_,…, *z*_*F*,*t*_}. In order to calculate weight of each particle, we need to do data association between state particle set of each object and the current observation *Z*_*t*_, which is shown by incidence matrix in the following formula:

(6) $$\begin{array}{c} C_{t}=\left[\begin{array}{cccc} c_{1,1} & c_{1,2} & \cdots & c_{1,N_{t}}\\ c_{2,1} & c_{2,2} & \cdots & c_{2,N_{t}}\\ \vdots & \vdots & \cdots & \vdots \\ c_{F_{t},1} & c_{F_{t},2} & \cdots & c_{F_{t},N_{t}} \end{array}\right], \end{array}$$

where *C*_*t*_ represents incidence matrix of time *t*, which is *F*_*t*_ × *N*_*t*_ dimensional matrix, *F*_*t*_ indicates the number of object observations, *N*_*t*_ indicates the number of object states, element *c*_*ij*_ in the matrix represents correlation degree of the *i*th observation value at time *t*, and *z*_*i*,*t*_, with observation value of particle set *y*_*jt*_, correspond to the *j*th object state. The larger value indicates the greater correlation degree. *c*_*ij*_ is calculated as follows:

 (1) Calculate feature similarity sim_*ij*_:
  (7) simij=12πσ2exp⁡−d2yit,yjtk2σ2,k=1,2,…,P,
 where sim_*ij*_ represents the similarity between the *i*th object observation value and all the particles set features which correspond to the *j*th object state; *y*_*it*_ represents the feature vector of a certain feature of the *i*th object; *y*_*jt*_ represents feature vector of each particle from the set which corresponds to the *j* object state; *d*[*y*_*it*_, *y*_*jt*_] shows the intersection distance between two features vectors.
 (2) Obtain the value of *c*_*ij*_ according to the following formula:
  (8) $$\begin{array}{c} c_{ij}=\max\left(\;a\cdot sim_{ij}^{color}+b\cdot sim_{ij}^{LBP}\;\right), \end{array}$$
 where sim_*ij*_^color^ and sim_*ij*_^LBP^ are, respectively, similarity function of color and LBP feature. 0 ≤ *a*, *b* ≤ 1 are weights of two feature likelihood functions. And they continuously carry out dynamic changes during particle transferring process. Weights *a* and *b* are, respectively, calculated as follows:
  (9) a=wcolorwcolor+wLBP,b=wLBPwcolor+wLBP,a+b=1,wcolor=max⁡simijcolor−min⁡simijcolor,wLBP=max⁡simijLBP−min⁡simijLBP,
 where sim_*ij*_^color^ and sim_*ij*_^LBP^, respectively, represent the weight of color features of all the particles and LBP feature at time *t*. *w*_color_ and *w*_LBP_ are distribution range of particle weights in each feature at time *t*.

Thus, we can get correlation condition between object observation value and particles set of object status according to the correlation matrix. Moreover, the appearance and disappearance of the object can also be derived from the analysis of the correlation matrix. If elements in a certain row of the correlation matrix are approximately zero, which indicates that no object status corresponds to the observed value of the time, it can be determined that the observation value is the newly emerging object. If elements in a certain line of the correlation matrix are approximately zero, which indicates that no observed value corresponds to a certain object status, it can be determined that the object of this status has disappeared.

#### 4.2.3. Determination of Object Disappearing and Appearing

According to the correlation matrix, we can know whether a certain object disappears or appears at time *t*. If object *j* in the correlation matrix is determined to have disappeared for three consecutive frames from time *t*, it can be determined that object *j* has disappeared. At this time, delete all particles in this status from the entire particle state. Then we get particle set as follows:

(10) $$\begin{array}{c} S_{t}=\left[\begin{array}{ccccccc} x_{1,t}^{1} & x_{2,t}^{1} & \cdots & x_{j-1,t}^{1} & x_{j+1,t}^{1} & \cdots & x_{N_{t},t}^{1}\\ x_{1,t}^{2} & x_{2,t}^{2} & \cdots & x_{j-1,t}^{2} & x_{j+1,t}^{2} & \cdots & x_{N_{t},t}^{2}\\ \vdots & \vdots & \cdots & \vdots & \vdots & \cdots & \vdots \\ x_{1,t}^{P} & x_{2,t}^{P} & \cdots & x_{j-1,t}^{P} & x_{j+1,t}^{P} & \cdots & x_{N_{t},t}^{P} \end{array}\right]. \end{array}$$

If object *k* in the correlation matrix is determined to have appeared for three consecutive frames from the time *t*, then it can be determined that object *k* is the newly appearing object. Then we get particle set as follows:

(11) $$\begin{array}{c} S_{t}=\left[\begin{array}{ccccc} x_{1,t}^{1} & x_{2,t}^{1} & \cdots & x_{N_{t},t}^{1} & x_{N_{t}+1,t}^{1}\\ x_{1,t}^{2} & x_{2,t}^{2} & \cdots & x_{N_{t},t}^{2} & x_{N_{t}+1,t}^{2}\\ \vdots & \vdots & \cdots & \vdots & \vdots \\ x_{1,t}^{P} & x_{2,t}^{P} & \cdots & x_{N_{t},t}^{P} & x_{N_{t}+1,t}^{P} \end{array}\right]. \end{array}$$

### 4.3. Occlusion Handling

**Figure 8:**
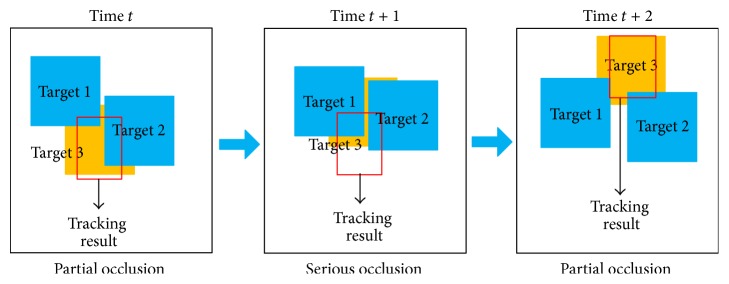
Tracking results under severe occlusion.

**Figure 9:**
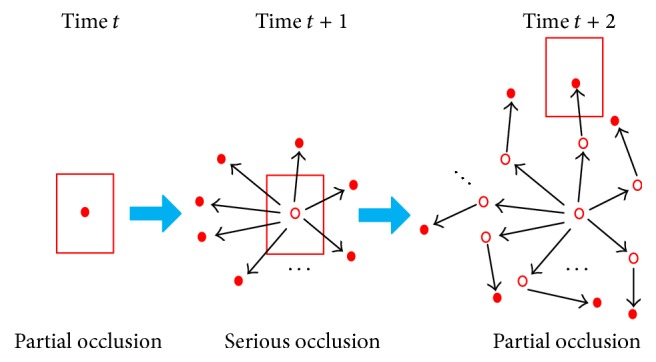
Movements of particles under severe occlusion.

In the process of tracking multiple pedestrians, mutual occlusion often occurs especially when object pedestrians are in serious occlusion. Useful pedestrians information cannot be extracted; thus it is likely to cause obscured pedestrians off-tracking results. The above tracking process can handle partial occlusion. However, when the object is in severe or complete occlusion, the value of likelihood function of predicted particles state will become very small. In view of this situation, the paper processes severe occlusion. When the entire likelihood function of particle state is smaller than a certain threshold, keep the last state of object tracking unchanged and the particles continue to do state transfer, since the object position of two adjacent frames differs less. However, when the object is severely occluded for more frames, in this paper, it will be determined as the object that disappeared. Object tracking results under severe occlusion as well as the movement of particles are, respectively, shown in Figures 8 and 9.

The advantage of this method is that when a pedestrian is determined as being in serious occlusion, although the object tracking results remain unchanged, the particle states continue to transfer in each frame. When the object reappears after several successive frames, it remains to be in the particle sampling range. Estimate the most likely candidate objects based on the candidate objects obtained by particles sampling at this time.

### 4.4. The Algorithmic Process

The entire algorithmic process can be summarized as in Algorithm 1.

## 5. Experimental Verification and Analysis

In order to evaluate the performance and tracking effect of proposed tracking algorithm, video sequences of real scenes are used to test the algorithm. Video data used in the test come from our database, CAVIAR database, and PETS2012 standard library. These video sequences include complex situations such as random translation, occlusion, scale change, likeness interference, and disappearance and appearance of the object.

Among them, parameter settings of the proposed tracking algorithm are as shown in Table 1; these parameters apply to all of the following test video data.

In the tracking process, root mean square error and average root mean square error are usually employed to evaluate the performance of the tracking algorithm; the root mean square error of time *t*  PositionError_*t*_ is as shown as follows:

(12) $$\begin{array}{c} PositionError_{t}=\sqrt{{\left(x_{t}^{\prime }-x_{t}\right)}^{2}+{\left(y_{t}^{\prime }-y_{t}\right)}^{2}}. \end{array}$$

Whereas (*x*_*t*_′, *y*_*t*_′) is the estimated value of the target position at time *t*, (*x*_*t*_, *y*_*t*_) is the real object position at time *t*.

The average RMS error is defined as

(13) $$\begin{array}{c} \bar{PositionError}=\frac{1}{Frames}\overset{Frames}{\underset{i=1}{\sum }}PositionError_{i}. \end{array}$$

Whereas Frames is the total number of tracked video sequence frames, $\bar{PositionError}$ is known as the average root mean square error, which is seen as a measurement of test result error; smaller value indicates better tracking effect.

### 5.1. Contrast Analysis

For comparison purpose, we compare the performance and tracking effect in these video sequences of BPF tracking algorithm [9], superpixel tracking algorithm [14], and the proposed tracking algorithm using the same video sequence. Reasons of comparison are as follows: (1) superpixel tracking algorithm mainly uses superpixel method to convert more pixels into fewer pixels to achieve tracking, whereas proposed tracking algorithm also uses superpixel to establish prior knowledge in the detection phase thus to achieve multiple pedestrian detection; (2) BPF tracking algorithm employs AdaBoost to detect pedestrians and particle filter algorithm to track pedestrians, whereas proposed tracking algorithm obtains confidence map of each frame by established prior knowledge and achieves pedestrian detection by random sampling of confidence map and therefore uses particle filter to achieve tracking. Both of them detect pedestrians at first and then achieve tracking by particle filter. Thus, there is certain similarity between them in the framework of the algorithm. Therefore, there is comparability among superpixel tracking algorithm, BPF tracking algorithm, and proposed tracking algorithm. Video sequence parameters for testing under the actual scene are shown in Table 2.

**Figure 10:**
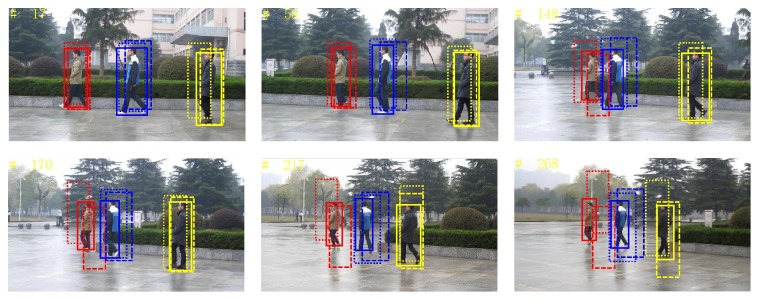
Sequence 1: tracking results. The results by our algorithm, superpixel tracking, and BPF methods are represented by solid line, dashed line, and dotted line rectangles. Rectangles in different colors denote the tracking results of different pedestrians.

*(1) Video Sequence 1*. This group of video sequence tests the tracking performance of proposed algorithm in the mobile background. It can be seen from Figure 10 that the background of video sequence is constantly changing during tracking process. Because neither superpixel tracking algorithm nor BPF tracking algorithm processes interference of objects according to background change, tracking drift and even tracking error occur during tracking procedure, as shown in frames 170, 217, and 268. Changes in background cause no impact on proposed algorithm, which can still track object stably. This is mainly due to accurate detection of the object for the first *M* frames, which results in more accurate tracking.

**Figure 11:**
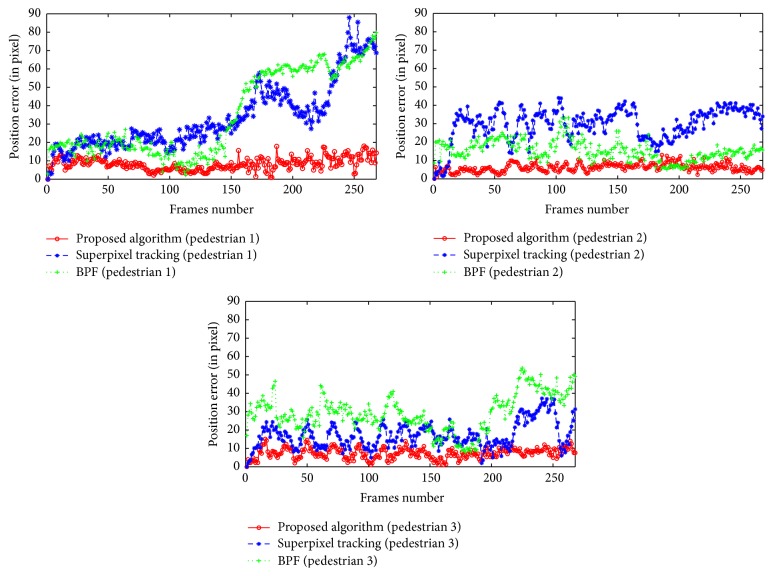
Sequence 1: pedestrians' error curves.

Figure 11 is the error curve which shows the error between three pedestrians in the process of tracking and the actual position, from left to right, is, respectively, the position error of tracking result in each frame for pedestrians 1, 2, and 3. It can be seen from the figure that, compared with the proposed algorithm, the position errors of superpixels tracking algorithm and BPF tracking algorithm are relatively large. Although pedestrian block does not appear in the video sequence, dynamic changes of the background cause a certain influence on the tracking results of the two algorithms, whereas the position error curve shows that proposed tracking algorithm has better accuracy.  Table 3 shows the average root mean square error of three algorithms' tracking results. It can be seen that the average root mean square error of proposed tracking algorithm is smaller, which indicates better tracking accuracy.

**Figure 12:**
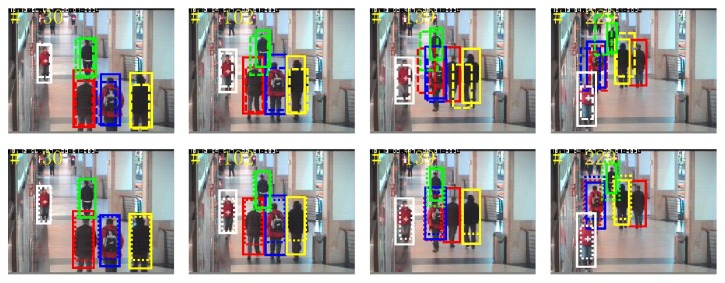
Sequence 2: tracking results. The results by our algorithm, superpixel tracking, and BPF methods are represented by solid line, dashed line, and dotted line rectangles. Rectangles in different colors denote the tracking results of different pedestrians.

*(2) Video Sequence 2*. The video data are from CAVIAR database. There have been two instances of serious occlusion during tracking this group of video sequences, and Figure 12 is a comparison among the results of three tracking algorithms. We can see that the proposed tracking algorithm can track the object and has a better tracking performance than the other two kinds of tracking algorithm.

**Figure 13:**
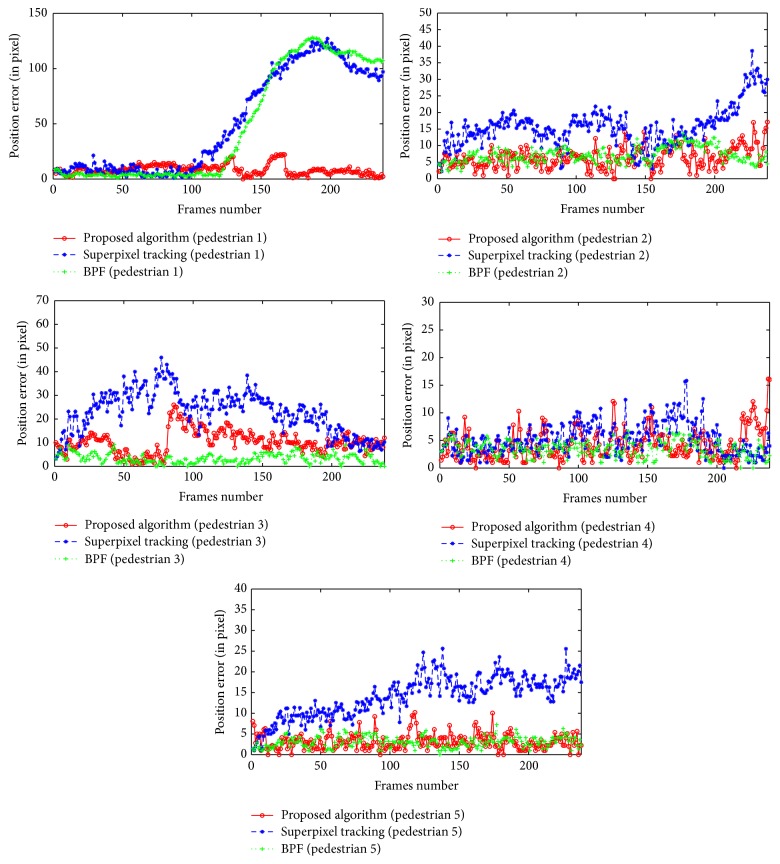
Sequence 2: pedestrians' error curves.

Figure 13 shows pedestrian error curves obtained by three tracking algorithms. It can be seen that superpixel tracking algorithm and the BPF tracking algorithm have larger tracking error for pedestrian 1, and BPF tracking algorithm has relatively small tracking error for pedestrians 3, 4, and 5, which indicates that BPF has better performance when there is no occlusion, whereas proposed tracking algorithm has relatively small root mean square error for all pedestrians. The average root mean square error in Table 4 indicates that the proposed tracking algorithm has better tracking accuracy in the tracking process.

**Figure 14:**
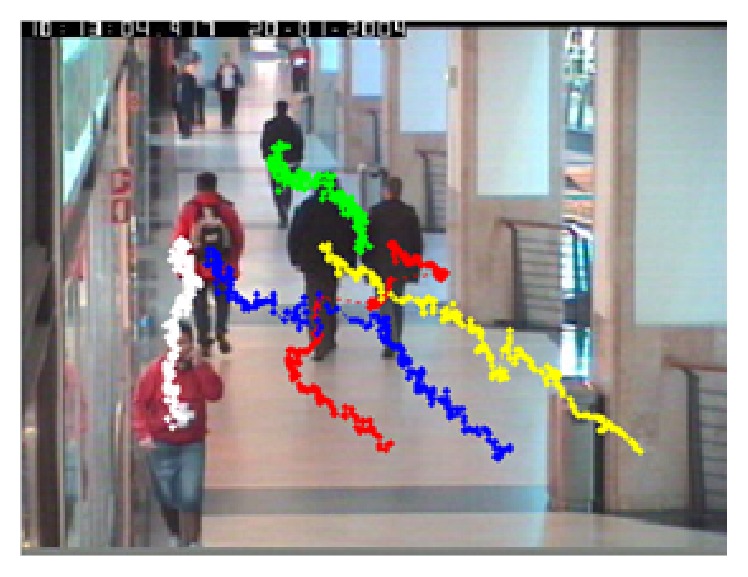
Sequence 2: object motion trajectories.

Figure 14 shows motion trajectories of five pedestrians tracked by proposed tracking algorithm, in which curves of different colors represent different pedestrian trajectories, and each dot represents coordinate position of pedestrian in each frame.

**Figure 15:**
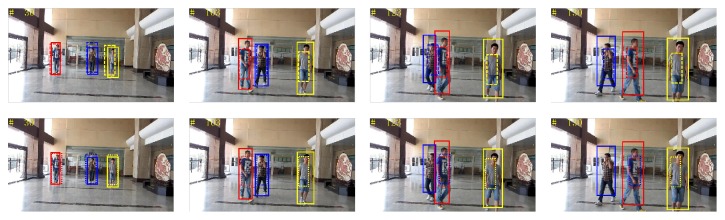
Sequence 3: tracking results. The results by our algorithm, superpixel tracking, and BPF methods are represented by solid line, dashed line, and dotted line rectangles. Rectangles in different colors denote the tracking results of different pedestrians.

*(3) Video Sequence 3*. The video shows three pedestrians walking in the hall, which aims to test the tracking effect of the three algorithms at the time of serious occlusion and changes in pedestrian scale.  Figure 15 shows pedestrians tracking results of the three methods. The first line shows tracking results of superpixel tracking algorithm and proposed tracking algorithm, while the second line indicates the tracking results of BPF tracking algorithm and proposed tracking algorithm. As can be seen from the figure, using BPF tracking algorithm, drift appears when there is severe pedestrian occlusion, such as frames 1, 2, and 3, thus affecting the accurate tracking of subsequent frames. The algorithm cannot acquire enough pedestrian features description when severe occlusion occurs and therefore easily causes failing of the tracking, whereas the superpixel tracking algorithm and the proposed algorithm can track the object by acquiring partial features and can handle severe occlusion; therefore they can get more accurate tracking results in the video sequence. However, due to the fact that BPF tracking algorithms and superpixel tracking algorithm do not deal with changes of the pedestrian scale, the tracking area is of a fixed size, while proposed tracking algorithm takes into account pedestrian scale changes in the state space model; thus it has better tracking effects compared with the other two tracking algorithms.

**Figure 16:**
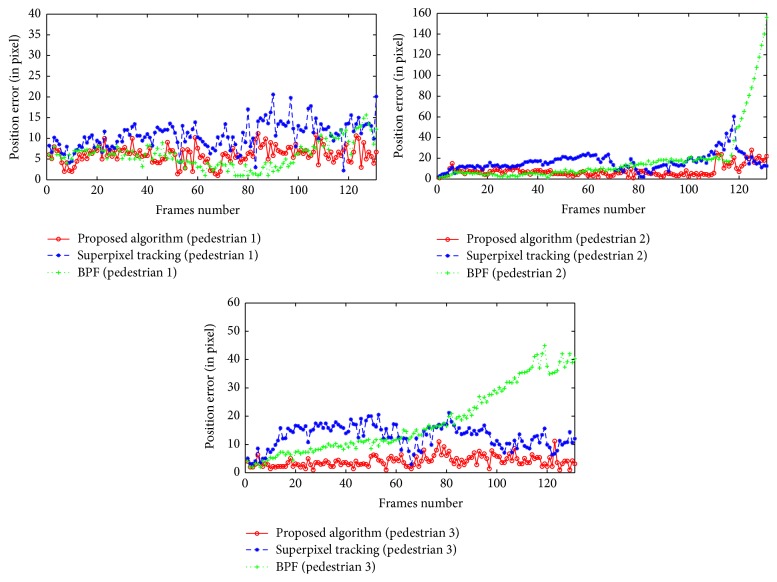
Sequence 3: pedestrians' error curves.

Figure 16, from left to right, respectively, shows the error curve of pedestrians 1, 2, and 3 between the tracking result and actual position in each frame. Red represents error curve of proposed tracking algorithm. It can be seen that, compared to the other two algorithms, proposed tracking algorithm has better accuracy and robustness. The average root mean square error in Table 5 shows that proposed tracking algorithm has better tracking accuracy in the video sequence.

**Figure 17:**
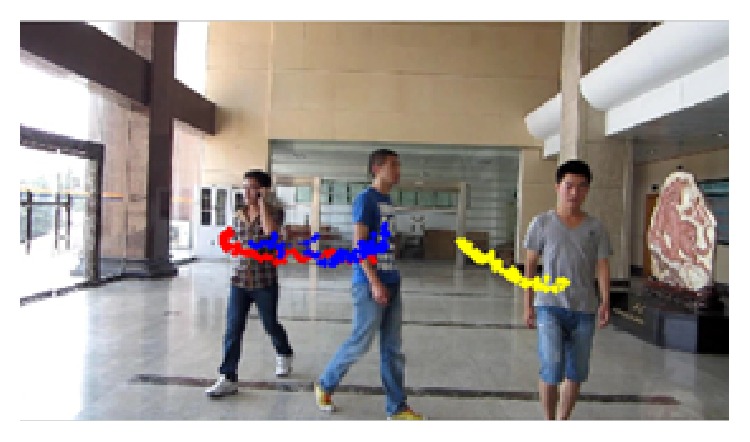
Sequence 3: object motion trajectories.

Figure 17 shows the target trajectories of three pedestrians, which are drawn by acquiring the coordinate positions of three pedestrians from the first frame to the last frame using proposed tracking algorithm.

**Figure 18:**
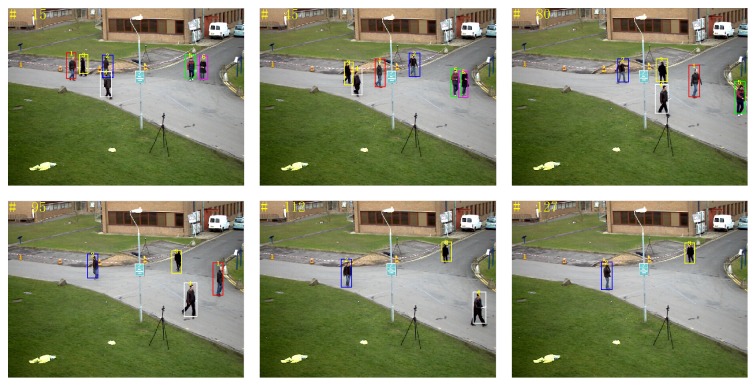
Tracking result: disappearance of objects.

*(4) Video Sequence 4*. The group of video sequences comes from PETS2012 standard library, mainly testing the impact of disappearance and appearance of objects on the tracking results. When an object has disappeared or appeared more than three times, it is considered that the object disappears or appears. In Figure 18, the rectangular frames in different colors represent different tracked pedestrians. At the 15th frame and the 45th frame, there are totally six tracked pedestrians; due to the limited range of the camera shot, pedestrians 6, 5, 1, and 4 disappear successively. As can be seen, proposed tracking algorithm can more accurately determine disappearance of the object.

**Figure 19:**
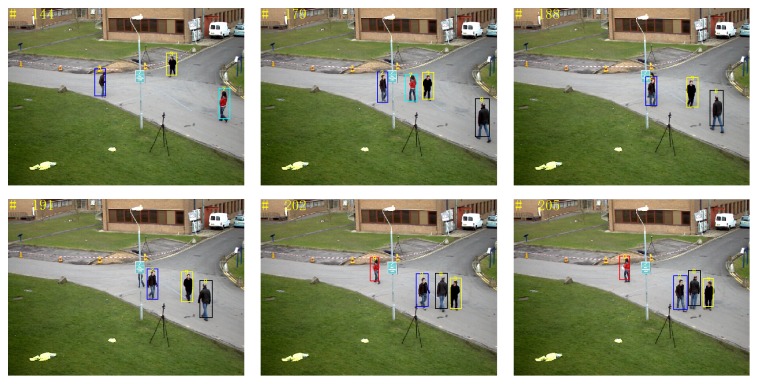
Tracking result: appearance of objects.

Figure 19 shows the tracking results of objects appearance by using proposed tracking algorithm. For instance, pedestrian 7 and pedestrian 8 appear in the scene in turn; proposed tracking algorithm accurately determines the appearance of objects. In the tracking process, pedestrian 7 is continuously severely blocked by pedestrian 2 and objects, and therefore at the 188th frame and 191st frame, pedestrian is considered to have disappeared. Because pedestrian 7 has disappeared more than three times, when it appears again (e.g., frame 202), it is considered as a new object and is given the new number 9.

**Figure 20:**
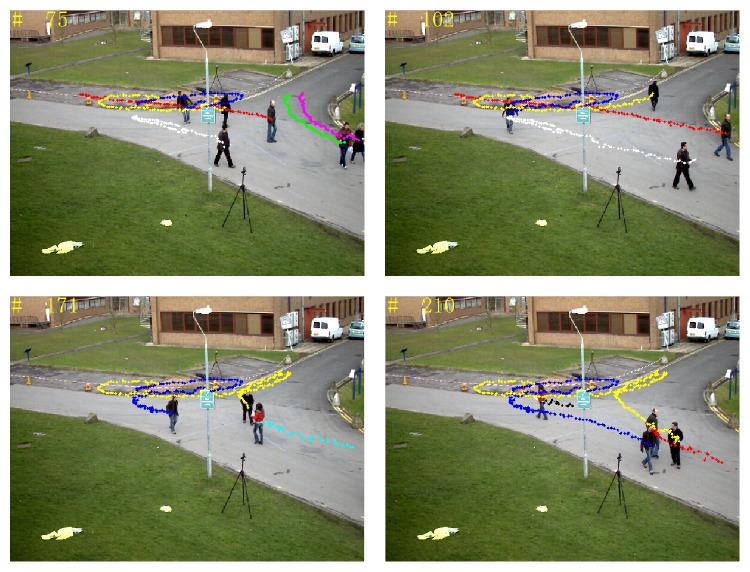
Multipedestrian tracking trajectories.

Figure 20 shows tracking trajectories of all pedestrians from the first frame to the current frame. Different colors represent different pedestrian trajectories, which provide an important basis for senior visual study such as pedestrian behavior recognition and scene understanding, and so forth.

## 6. Conclusion

In order to solve multipedestrian tracking problems in video sequences, this paper proposes tracking algorithm of multiple pedestrians based on particle filters. The contributions of our work can be listed as follows: (1) we apply frame difference and HOG to getting object regions in training frames rapidly and accurately and acquire object and background confidence by establishing a priori knowledge to track pedestrians; (2) we integrate color and texture features into particle filter to get better observation results and then automatically adjust the weight value of each feature according to the current tracking environment; (3) we correlate the test results with the particle states and propose discrimination method for object disappearance and appearance, thus achieving multipedestrian tracking in complex scenarios. Test results show that proposed algorithm has better stability and robustness in complex situations such as moving background, pedestrian translation, severe occlusion, interference among pedestrians, pedestrian scale change, and disappearance and appearance of pedestrians. However, there is still a lack of a common tracking algorithm for various kinds of complex environments, such as pedestrian tracking in moving objects. Therefore, improvements and amendments are still needed in future studies.

## Figures and Tables

**Algorithm 1:**
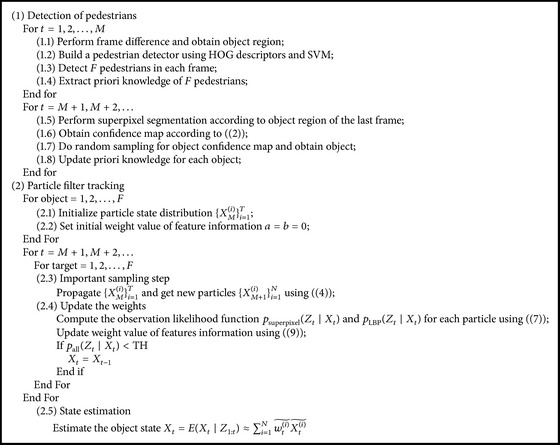
The entire algorithmic process.

**Table 1:** Parameter settings of proposed tracking algorithm.

Parameter	Value
Number of training frames	5
Number of superpixel blocks	200
Number of particles	300
Number of frames determining disappearance and appearance of the object	3

**Table 2:** Parameters of test video sequences.

Video sequence	Frame size	Total frames	Frame speed (fps)
(1) Three pedestrians in dynamic background	800 × 450	268	30
(2) Five pedestrians in the corridor	384 × 288	238	25
(3) Three pedestrians in the hall	800 × 450	131	30
(4) Sparse crowd	768 × 576	210	30

**Table 3:** Sequence 1: average root mean square error.

	Superpixel tracking algorithm	BPF tracking algorithm	Proposed algorithm
Pedestrian 1	33.4929	35.1872	8.2831
Pedestrian 2	29.9134	15.3953	6.187
Pedestrian 3	17.2689	29.447	7.3893

**Table 4:** Sequence 2: average root mean square error.

	Superpixel tracking algorithm	BPF tracking algorithm	Proposed algorithm
Pedestrian 1	51.0719	48.0748	8.3892
Pedestrian 2	15.6293	7.1292	6.3603
Pedestrian 3	23.0312	3.4245	10.3213
Pedestrian 4	5.2422	3.4314	4.2821
Pedestrian 5	14.0239	3.0628	3.1802

**Table 5:** Sequence 3: average root mean square error.

	Superpixel tracking algorithm	BPF tracking algorithm	Proposed algorithm
Pedestrian 1	10.8409	5.9901	6.0895
Pedestrian 2	16.5392	18.5687	7.8006
Pedestrian 3	12.8657	18.086	4.0837

## References

[1] Sui Y., Zhang L. (2015). Visual tracking via locally structured Gaussian process regression. *IEEE Signal Processing Letters*.

[2] Zhou T., He X., Xie K., Fu K., Zhang J., Yang J. (2015). Robust visual tracking via efficient manifold ranking with low-dimensional compressive features. *Pattern Recognition*.

[3] Li H., Liu Y., Xiong S., Wang L. (2014). Pedestrian detection algorithm based on video sequences and laser point cloud. *Frontiers of Computer Science*.

[4] Führ G., Jung C. R. (2014). Combining patch matching and detection for robust pedestrian tracking in monocular calibrated cameras. *Pattern Recognition Letters*.

[5] Hwang J. P., Baek J., Choi B., Kim E. (2015). A novel part-based approach to mean-shift algorithm for visual tracking. *International Journal of Control, Automation and Systems*.

[6] Yu W., Tian X., Hou Z., Zha Y., Yang Y. (2015). Multi-scale mean shift tracking. *IET Computer Vision*.

[7] Xu J., Deng W. (2015). A robust online learning method for object tracking. *Journal of Information and Computational Science*.

[8] Fiaschi L., Diego F., Gregor K. Tracking indistinguishable translucent objects over time using weakly supervised structured learning.

[9] Li G., Jiang S., Zhang W., Pang J., Huang Q. (2016). Online web video topic detection and tracking with semi-supervised learning. *Multimedia Systems*.

[10] Carmi A. Y., Mihaylova L., Septier F. (2016). Subgradient-based Markov Chain Monte Carlo particle methods for discrete-time nonlinear filtering. *Signal Processing*.

[11] Varas D., Marques F. Region-based particle filter for video object segmentation.

[12] Choe G., Wang T., Liu F., Choe C., So H. (2015). Visual tracking based on particle filter with spline resampling. *Multimedia Tools and Applications*.

[13] Isard M., MacCormick J., Brambl E. A Bayesian multiple-blob tracker.

[14] Okuma K., Taleghani A., Freitas N. A boosted particle filter: multitarget detection and tracking.

[15] Henriques J. F., Caseiro R., Martins P., Batista J. Exploiting the circulant structure of tracking-by-detection with kernels.

[16] Danelljan M., Khan F. S., Felsberg M., Van De Weijer J. Adaptive color attributes for real-time visual tracking.

[17] Lu W.-L., Ting J.-A., Murphy K. P., Little J. J. Identifying players in broadcast sports videos using conditional random fields.

[18] Chen S., Fern A., Todorovic S. Multi-object tracking via constrained sequential labeling.

[19] Yang F., Lu H., Yang M.-H. (2014). Robust superpixel tracking. *IEEE Transactions on Image Processing*.

[20] Xue H., Liu Y., Cai D., He X. (2016). Tracking people in RGBD videos using deep learning and motion clues. *Neurocomputing*.

[21] Wu G., Lu W., Gao G., Zhao C., Liu J. (2016). Regional deep learning model for visual tracking. *Neurocomputing*.

